# More than skin-deep: visceral fat is strongly associated with disease activity, function and metabolic indices in psoriatic disease

**DOI:** 10.1186/s13075-023-03085-9

**Published:** 2023-06-23

**Authors:** Tim Blake, Nicola J. Gullick, Charles E. Hutchinson, Abhir Bhalerao, Sarah Wayte, Andrew Weedall, Thomas M. Barber

**Affiliations:** 1 Department of Rheumatology, University Hospitals Coventry and Warwickshire, Clifford Bridge Road, Coventry, CV2 2DX UK; 2 Warwick Medical School, University of Warwick, Coventry, CV4 7HL UK; 3 Division of Biomedical Sciences, Warwick Medical School, Clinical Sciences Research Laboratories, University Hospitals Coventry and Warwickshire, Coventry, CV2 2DX UK; 4 Department of Computer Science, University of Warwick, Coventry, CV4 7EZ UK; 5 Department of Radiology, University Hospitals Coventry and Warwickshire, Coventry, CV2 2DX UK; 6 Radiology Physics, Department of Clinical Physics and Bioengineering, University Hospitals Coventry and Warwickshire, Coventry, CV2 2DX UK; 7 Warwickshire Institute for the Study of Diabetes, Endocrinology and Metabolism, University Hospitals Coventry and Warwickshire, Coventry, CV2 2DX UK

**Keywords:** Psoriasis, Psoriatic arthritis, Psoriatic disease, Body composition, Central fat distribution, Health-related quality of life, Metabolic syndrome

## Abstract

**Objective:**

To compare body composition between patients with psoriatic disease (PsD), including cutaneous psoriasis (PsO) and psoriatic arthritis (PsA), and controls, and to explore associations between disease activity and measures of function and metabolic derangement.

**Methods:**

Body composition was assessed by air displacement plethysmography (ADP) and MRI-derived fat segmentation using an automated pipeline (FatSegNet). Function was assessed by Health Assessment Questionnaire (HAQ) and metabolic status by fasting lipid profile, insulin and adiponectin. Active and inactive PsO and PsA were defined by body surface area (BSA) and Psoriasis Area Severity Index (PASI) and minimal disease activity (MDA), respectively.

**Results:**

Thirty patients (median disease duration 15 years; median age 52 years) and 30 BMI-matched controls were enrolled. Compared with controls, all MRI-derived body composition parameters—whole-body volume, subcutaneous adipose tissue (SAT), visceral adipose tissue (VAT), abdominal adipose tissue (AAT), VAT/AAT and VAT/SAT—were higher in the PsD group, specifically, those with active disease. Body mass, body fat, whole-body volume and whole-body VAT were correlated with higher triglycerides, cholesterol:HDL (high-density lipoprotein), insulin resistance and lower adiponectin as well as higher HAQ and lower MDA.

**Conclusions:**

In this pilot study, patients with PsD revealed excessive total adipose tissue and a greater volume of metabolically unfavourable ectopic fat, including VAT, compared with BMI-matched controls, which also correlated with HAQ, disease activity and overall dysmetabolism. We also provide the first evidence in patients with PsD for the clinical application of FatSegNet: a novel, automated and rapid deep learning pipeline for providing accurate MRI-based measurement of fat segmentation. Our findings suggest the need for a more integrated approach to the management of PsD, which considers both the metabolic and inflammatory burden of disease. More specifically, visceral fat is a surrogate marker of uncontrolled PsD and may be an important future target for both pharmacological and lifestyle interventions.

**Supplementary Information:**

The online version contains supplementary material available at 10.1186/s13075-023-03085-9.

## Introduction

Psoriasis is an immune-mediated chronic inflammatory disease affecting the skin, entheses and joints, with an estimated prevalence in adults ranging from 0.5 to 11.4%, and in children from 0 to 1.4% [1]. Psoriatic disease (PsD) is an umbrella term used to encompass the multitude of ways that psoriasis can manifest clinically, including both cutaneous psoriasis (PsO) and psoriatic arthritis (PsA) [2].

Obesity commonly co-exists with PsO and PsA and contributes to both the onset and severity of PsD, possibly through enhancement of inflammatory processes [3–5]. This association is shared with metabolic syndrome (MetS), not least the increased prevalence of cardiovascular (CV) risk factors and the ensuing CV morbidity [6–10]. Recent studies have suggested that adipokines such as leptin, adiponectin and resistin, produced by adipocytes and dysregulated in obesity and metabolic syndrome, as well as tumour necrosis factor alpha (TNF-α), a multifunctional cytokine and adipokine, are key mediators that link obesity and its associated chronic inflammatory milieu with the pathogenesis of PsD. The onset and development of PsD, by way of a common inflammatory pathway, may give rise to vascular inflammation, atherosclerosis and thrombosis, resulting in exaggerated morbidity [11]. Accordingly, adipose tissue in the context of obesity, through its release of local and systemic factors (including adipokines), has the capacity to induce a low-level chronic inflammatory state that can have important knock-on consequences for the onset and development of inflammatory conditions such as PsD [12].

Preliminary data suggest that PsO and PsA associate with a metabolically unfavourable body composition [13]. However, the existing literature on PsD lacks clear-cut data on body composition, including fat-free tissue (lean body mass) and fat tissue in eutopic (subcutaneous and visceral) and ectopic (hepatic and myocellular) depots. Furthermore, the role of changes in body composition as a possible causative factor in the pathogenesis of PsD, as opposed to a phenotypic non-causative feature of PsD or even a feature that is only related to PsD through some other common factor(s), remains incompletely understood. Body composition, a collective term for lean and fat mass proportions, provides a useful indicator of metabolic health, and its assessment in patients with PsD could provide invaluable insight into overall cardiometabolic risk that in turn could inform important decisions regarding ongoing clinical management [14].

Regarding the assessment of body composition in chronic diseases such as PsD, there is a paucity of data in the existing literature on the use of MRI techniques, with reports tending to focus on other less widely available modalities such as air displacement plethysmography (ADP), dual energy X-ray absorptiometry (DXA), bioimpedance analysis (BIA) and computed tomography (CT), as highlighted in a recent systematic review [15]. Despite its under-representation in the literature, MRI has the capacity to provide accurate measures of total body adipose tissue and to clearly delineate adipose tissue depots (including those in subcutaneous and visceral locations). Different compartments of adipose tissue are associated with distinctive pathophysiological effects and resultant morbidity, principally CV disease, glucose impairment and dyslipidaemia [16–19].

Recent studies also indicate a correlation between the accumulation of visceral adipose tissue and adverse metabolic and inflammatory profiles [20, 21]. Therefore, MRI seems an attractive imaging option for research and clinical purposes. MRI-determined fat measurements have shown high correlation with those obtained by ADP [22]. As such, MRI techniques enable automated, accurate localisation and segmentation of adipose tissue depots, which is often invaluable for research into metabolic diseases [23]. Despite its clear benefits and advantages, however, no reported studies have included validated MRI-based adipose tissue measurement systems for patients with PsD compared to controls. To address this important unmet need, our aim was to implement and validate an MRI-based protocol to accurately quantify body composition in PsD vs. BMI-matched controls. Further aims included comparisons with ADP measures of body composition and to correlate measures of body composition with PsD disease activity and markers of metabolic status.

## Methods

### *BODYCOPAstudy design*

BODYCOPA (BODY COmposition in PsoriAtic disease) was a single-centre, cross-sectional, pilot study conducted at University Hospitals Coventry and Warwickshire (UHCW) NHS Trust between August 2021 and June 2022, supported by the Human Metabolism Research Unit (HMRU) and the University of Warwick. BODYCOPA was designed to assess the metabolic and body composition profiles of patients with PsD. Patients were recruited from dermatology and rheumatology outpatient clinics at UHCW and included those with an existing diagnosis of PsO (all subtypes) and PsA, on conventional topical and non-targeted/non-biological medications. All PsA patients fulfilled the “Classification Criteria for Psoriatic Arthritis” (CASPAR) standards [24]. Recruited participants remained on their selected management (clinically agreed and confirmed) for the duration of the study. We excluded from recruitment any patient < 18 years of age. Other exclusion criteria were pregnancy, systemic corticosteroids or biological drugs for any condition, renal disease, diabetes mellitus, intensive physical training or dieting, any patient on levothyroxine without a stable TSH within the normal range, any patient on cholesterol-lowering therapy, claustrophobia or inability to undergo an MRI scan for 30 min. Patients with obesity and hypertension as well as patients with hypothyroidism on levothroxine replacement were included, provided they had a serum TSH demonstrating biochemical euthyroidism. Healthy controls were recruited following public advertisement at the study site, whilst matching to recruited patient participants for BMI and ethnicity.

### Acquisition of anthropometric and clinical data

Each recruited participant had anthropometric assessment of body weight, height, BMI and waist:hip ratio. Body weight (kg) and height (m) were measured by trained research nurses with the participant in light clothes and without shoes. BMI was calculated as weight (kg)/height (m^2^). Waist circumference (cm) was measured at the midpoint between the lower margin of the last palpable rib and the top of the iliac crest. Waist:hip ratio was calculated as the waist circumference divided by the hip circumference. Clinical assessments—PASI, Dermatology Life Quality Index (DLQI), Disease Activity in Psoriatic Arthritis (DAPSA) and MDA—were performed by a trained consultant rheumatologist and designated research nurses. All recruited participants completed the validated and widely accepted International Physical Activity Questionnaire (IPAQ)—long-version comprising 5 activity domains—to provide data on health-related physical activity [25]. Composite disease activity of PsD, referred to as ‘overall activity’, was calculated from combined skin (BSA and PASI) and MDA scores and defined as ‘MDA not achieved’ and/or ‘moderate’ (BSA 3–10% or PASI 5–10) or ‘severe’ (BSA > 10% or PASI > 10) PsO.

### Laboratory analyses

Fasting serum samples were stored initially at − 20° C and then transferred to a − 80° C freezer prior to biochemical analysis. Metabolic indices included lipid profile, high-sensitivity CRP, glucose, insulin and adiponectin. HOMA-IR (homeostasis model assessment for insulin resistance) was calculated for all participants.

### Body composition (ADP)

Body fat was assessed for each participant using ADP (a whole-body densitometric technique based on air displacement) within a BODPOD® (Life Measurement Inc, USA.) body composition system, housed within HMRU at UHCW. Participants were advised, in line with the manufacturer’s instructions, to wear tight-fitting clothing or swimwear and swimming cap and to avoid food, drink or exercise for at least 3 h prior to testing.

### Body composition (MRI)

Each participant had a whole-body axial MRI scan acquired on a 3.0 T GE Discovery™ MR750wscanner. A 3D LAVA-Flex sequence with 8 mm slice width interpolated to 4 mm and 2 mm by 2 mm in-plane resolution. Water, fat and in-phase images were acquired. We used FatSagNet software (https://deep-mi.org/research/fatsegnet/) to derive adipose tissue segmentation from the Dixon MR images. FatSegNet is a novel, rapid and fully automated computer software system and deep learning pipeline employed to accurately identify and quantify VAT and SAT within anatomically pre-defined abdominal regions, reported as volume (cm^3^) [26]. In accordance with FatSegNet, the abdominal region was divided into three different blocks: the abdominal region (from the lower boundary of T12 vertebra to the lower boundary of L5 vertebra), the thoracic region (everything above the lower boundary of T12 vertebra) and the pelvic region (everything below the lower boundary of L5 vertebra). Radiological data were transmitted, stored, retrieved and processed according to Digital Imaging and Communications in Medicine (DICOM) standards. All variables were extracted from the predicted segmentation maps of the FatSegNet pipeline.

### Statistical analyses

The target number of participants was based on a power calculation using data generated by other reported studies on PsD of similar design and outcomes [27]. All statistical analyses were performed using SPSS Statistics for Windows, version 27.0 (IBM, Armonk, NY, USA). Mean, median, S.D. and IQR were used to represent continuous variables where data were parametric. Comparisons between data from participants and matched controls were depicted by the chi-squared or Fisher’s exact test for categorical variables. For continuous variables, independent *t*-test or Mann–Whitney *U* test was performed for parametric and non-parametric data respectively.

Univariate and multivariate (adjusted for age and sex) linear regression analyses were performed to explore possible correlations between measures of PsD disease activity (including inflammatory and insulin resistance status) as independent variables and body composition parameters (including total body mass, body fat, fat-free mass, whole-body volume, whole-body VAT and whole-body VAT/SAT ratio) as dependent variables. Five explanatory variables considered clinically relevant correlates for total body mass including (i) body fat, (ii) fat-free mass, (iii) whole-body volume, (iv) whole-body VAT and (v) whole-body VAT/SAT ratio were then entered in a forward stepwise manner until a best-fit model was achieved. Data on the strength of these correlations were provided as the unstandardised *ꞵ* with 95% CIs. R-squared was calculated as a goodness-of-fit for each regression model. Pearson correlation coefficients were employed to depict covariance of body composition variables in the context of physical activity, disease activity, function and metabolic dysfunction. All tests were two-sided and a *P*-value < 0.05 was considered statistically significant.

## Results

### Baseline characteristics

Baseline data for the recruited participants with PsD (*n* = 30) are shown in Table 1. At recruitment, median PsD disease duration was 15.0 years (IQR 20). Recruited participants were predominantly female (*n* = 17; 56.7%) and white Caucasian ethnicity (*n* = 28; 93.3%). Diets were mainly non-restrictive (*n* = 26; 86.6%) and half the PsD participants had never smoked (*n* = 15). At baseline, although there was no significant difference in BMI between the PsD group and the BMI/ethnicity-matched control patients, waist:hip ratio was significantly greater in the PsD group. The two groups did differ in their ages (median age 52 [IQR 18] vs. 42 [IQR 19] years for PsD and control groups respectively; *P*-value < 0.05). Regarding treatments for PsD, most had regular topical therapy (*n* = 22, 73%) and a non-biologic systemic therapy (*n* = 20, 67%). Regarding severity of PsD, most had mild disease (*n* = 21, 70%), defined as BSA < 3% and/or PASI < 5 [28]. Furthermore, a majority (*n* = 14, 64%) of the PsD group with concurrent PsA did not achieve MDA, in whom the presence of tender and swollen joints and DAPSA scores were low, suggesting a state of low disease activity. However, MDA scores were counterbalanced by entheseal, VAS and HAQ scores which were modest, thereby enhancing the aggregate effect of overall PsD activity. PsD-derived data are summarised in Table 2.

**Table 1:** Baseline characteristics

	Total psoriasis (*n* = 30)	With PsA (*n* = 22)	Without PsA (*n* = 8)	Controls (*n* = 30)	*P*-value
Gender, *n* (%) females	17 (56.7)	9 (40.9)	8 (100.0)	22 (73.3)	0.279*
Age (years)					
Mean (SD)	50.03 (11.89)	49.82 (12.69)	50.63 (10.13)	42.33 (11.50)	0.007**
Median (IQR)	52.00 (18)	51.5 (21)	53 (16.5)	42.00 (19.0)	
Disease duration (years)					
Mean (SD)	18.31 (14.33)	13.24 (10.48)	32.25 (14.76)	NA	NA
Median (IQR)	15 (23)	10 (13)	34 (18.5)	NA	
Ethnicity, *n* (%)					
Asian/Asian British	2 (6.7)	1 (4.55)	1 (12.5)	3 (10.0)	0.324**
White	28 (93.3)	21 (95.45)	7 (87.5)	27 (90.0)	
BMI (kg/m^2^)					
Mean (SD)	29.59 (6.04)	28.86 (5.13)	31.59 (8.13)	29.57 (7.76)	0.495**
Median (IQR)	28.55 (7.98)	28.55 (6.3)	31.05 (10.2)	27.65 (10.4)	
W:H ratio					
Mean (SD)	0.94 (0.09)	0.95 (0.09)	0.91 (0.09)	0.87 (0.06)	0.001**
Median (IQR)	0.95 (0.12)	0.96 (0.09)	0.9 (0.13)	0.86 (0.05)	
Diet, *n* (%)					
Eat everything	26 (86.6)	18 (81.8)	8 (100.0)	26 (86.6)	0.500*
Vegetarian	2 (6.7)	2 (9.1)	0 (0.0)	2 (6.7)	
Flexitarian	2 (6.7)	2 (9.1)	0 (0.0)	2 (6.7)	
Pescetarian	0 (0.0)	0 (0.0)	0 (0.0)	0 (0.0)	
Smoking, *n* (%)					
Never	15 (50.0)	11 (50.0)	4 (50.0)	23 (76.6)	0.435*
Ex	12 (40.0)	9 (40.9)	3 (37.5)	5 (16.7)	
Current	3 (10.0)	2 (9.1)	1 (12.5)	2 (6.7)	
Metabolic syndrome					
Present	3 (10.0)	2 (9.09)	1 (12.5)	2 (6.7)	1.000*

Significant values are in italics

*BMI* body mass index, *W:H* waist/height

^*^Fisher’s exact test, **Independent *t*-test: psoriatic group vs. controls

**Table 2:** Overall characteristics of patients with psoriatic disease

Clinical characteristics	Value (*n* = 30)
Psoriasis history, *n* (%)	
Concomitant psoriatic arthritis	22 (73.3)
Nail	9 (30)
Plaque	26 (86.6)
Scalp	13 (43.3)
Guttate	0 (0.0)
Genital	1 (3.3)
Inverse	0 (0.0)
Pustular	0 (0.0)
Erythrodermic	0 (0.0)
Disease activity	
Disease duration (years)	
Mean (SD)	18.31 (14.08)
Median (IQR)	15.00 (23.0)
PASI	
Mean (SD)	2.17 (2.98)
Median (IQR)	0.45 (4.4)
BSA	
Mean (SD)	2.43 (3.96)
Median (IQR)	0 (3.0)
HAQ	
Mean (SD)	0.67 (0.66)
Median (IQR)	0.437 (1.375)
DLQI	
Mean (SD)	5.0 (6.68)
Median (IQR)	5.0 (9.0)
VAS Global	
Mean (SD)	54.1 (28.23)
Median (IQR)	56.5 (46.0)
VAS Pain	
Mean (SD)	38.05 (28.00)
Median (IQR)	27.5 (43.0)
Swollen joint count (66)	
Mean (SD)	1.36 (3.22)
Median (IQR)	0 (1.0)
Tender joint count (68)	
Mean (SD)	2.14 (5.02)
Median (IQR)	0 (1.0)
LEI	
Mean (SD)	0.91 (1.51)
Median (IQR)	0 (1.0)
SPARCC	
Mean (SD)	0 (2.70)
Median (IQR)	0 (2.0)
DAPSA	
Mean (SD)	13.36 (10.08)
Median (IQR)	12.5 (10.0)
PsO severity, n (%)	
Mild (BSA < 3% or PASI < 5)	21 (70.0)
Moderate (BSA 3–10% or PASI 5–10)	6 (20.0)
Severe (BSA > 10% or PASI > 10)	3 (10.0)
MDA	
7 (VLDA achieved)	1 (4.55)
5–7 (MDA achieved)	7 (31.81)
< 5 (MDA not achieved)	14 (63.64)
Overall activity^a^	
Not active	11 (36.67)
Active	19 (63.33)
Topical therapy, *n* (%)	
Glucocorticoid	21 (70.0)
Calcipotriol	1 (3.3)
Non-biologic systemic therapy, *n* (%)	
Phototherapy	12 (40.0)
Acitretin	5 (16.7)
Methotrexate	2 (6.7)
Sulfasalazine	1 (3.3)
Ciclosporin	0 (0.0)

*BSA* body surface area, *DAPSA* Disease Activity index for Psoriatic Arthritis, *DLQI* Dermatology Life Quality Index, *HAQ* Health Assessment Questionnaire, *IQR* interquartile range, *LEI* Leeds Enthesitis Index, *MDA* minimal disease activity, *PASI* Psoriasis Area and Severity Index, *PsO* psoriasis, *SD* standard deviation, *SPARCC* Spondyloarthritis Research Consortium of Canada, *VLDA* very low disease activity

^a^Overall activity defined as MDA not achieved and/or moderate or severe psoriasis

All group-specific outcomes including metabolic and body composition variables are shown in Supplementary Table 1.

### BODPOD®-derived body composition parameters

**Fig. 1:**
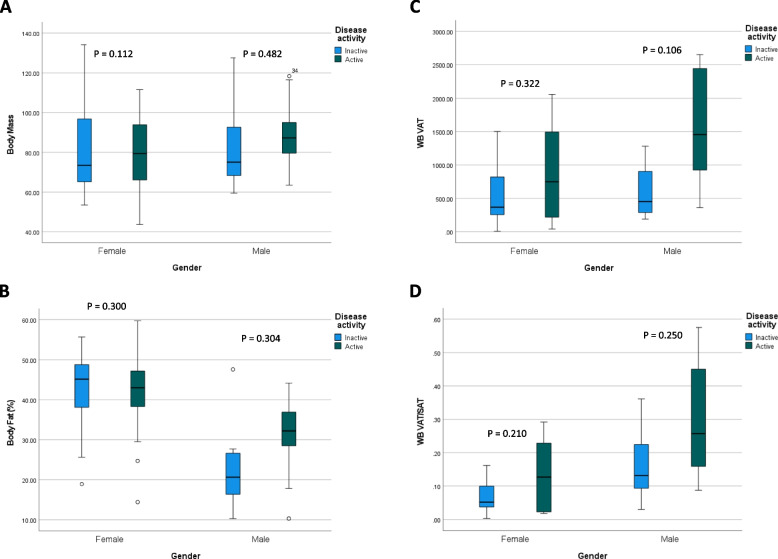
BODPOD®-derived body composition and MRI segmentation in patients with PsD according to sex and disease activity

BODPOD®-derived body composition measures in patients with PsD according to disease activity are summarised in Table 3 and illustrated in Fig. 1A and B. Overall, whole-body mass by ADP was 11.0% higher in the psoriatic group compared to BMI/ethnicity-matched controls, yet no difference was observed with respect to body fat.

**Table 3:** Body composition in patients with PsO and PsA according to disease activity

	PsO/A active (*n* = 19)	PsO/A inactive (*n* = 11)	*P*-value	Total PsO/A (*n* = 30)	Controls (*n* = 30)	*P*-value
BodPod						
Body mass (kg)						
Mean (SD)	87.64 (19.89)	74.50 (17.69)	0.046*	82.99 (19.80)	73.9 (20.14)	0.328*
Median (IQR)	87.3 (28.8)	77.8 (36.8)		83.35 (28.6)	73.9 (29.5)	
Body fat %						
Mean (SD)	35.71 (12.33)	38.95 (10.04)	0.233*	36.85 (11.47)	36.99 (13.37)	0.965*
Median (IQR)	37.4 (13.7)	41.5 (14.6)		37.85 (13.6)	40.85 (21.9)	
Fat mass (kg)						
Mean (SD)	33.23 (15.57)	32.47 (14.24)	0.448*	32.95 (14.85)	31.14 (16.52)	0.329*
Median (IQR)	35 (21.1)	29.6 (15.1)		31.4 (15.1)	28.2 (25.5)	
Fat-free mass (kg)						
Mean (SD)	54.38 (11.90)	48.3 (9.01)	0.077*	52.15 (11.17)	49.3 (10.71)	0.822*
Median (IQR)	52.8 (17.4)	44.9 (13.4)		50.2 (18.8)	47.65 (12.6)	
MRI						
WB volume						
Mean (SD)	21,824.11 (5025.65)	20,113.48 (3946.38)	0.180*	21,371.85 (4730.87)	20,113.49 (3946.38)	0.107*
Median (IQR)	22,259.19 (7699.27)	21,082.53 (4856.21)		22,259.19 (6308.91)	21,082.53 (4856.21)	
WB SAT						
Mean (SD)	6291.76 (2774.51)	5885.37 (1986.09)	0.342*	6133.47 (2502.11)	5885.37 (1986.09)	0.283*
Median (IQR)	6794.84 (3833.69)	5441.88 (2453.08)		6533.14 (3175.75)	5441.88 (2453.08)	
WB VAT						
Mean (SD)	1211.27 (771.34)	1131.46 (679.27)	0.393*	1202.45 (761.99)	1131.46 (679.27)	< 0.001*
Median (IQR)	1237.96 (1559.67)	1147.13 (879.19)		1202.82 (1142.86)	1147.13 (879.19)	
WB VAT/AAT						
Mean (SD)	0.23 (0.18)	0.19 (0.13)	0.296*	0.16 (0.11)	0.08 (0.06)	< 0.001*
Median (IQR)	0.18 (0.22)	0.19 (0.21)		0.15 (0.17)	0.06 (0.08)	
WB VAT/SAT						
Mean (SD)	0.22 (0.18)	0.19 (0.13)	0.296*	0.22 (0.17)	0.19 (0.13)	< 0.001*
Median (IQR)	0.18 (0.22)	0.18 (0.21)		0.18 (0.24)	0.18 (0.21)	

Significant values are in italics

*AAT* abdominal adipose tissue, *IQR* interquartile range, *SAT* subcutaneous adipose tissue, *SD* standard deviation, *VAT* visceral adipose tissue, *WB* whole-body

^*^Independent *t*-test

### MRI-derived body composition parameters

**Fig. 2:**
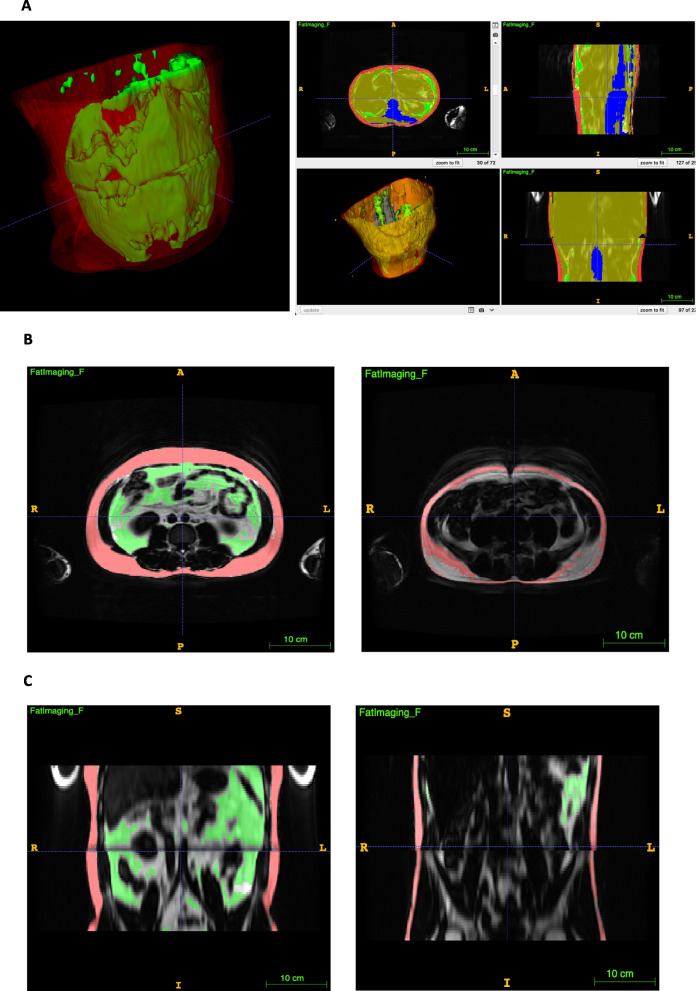
**A** 3D fat imaging. **B **Axial fat segmentation in a 32-year-old male patient compared to that of an age-, sex- and BMI-matched control (visceral fat depicted in green). **C** Coronal fat segmentation in a 32-year-old male patient compared to that of an age-, sex- and BMI-matched control (visceral fat depicted in green)

Similar to the BODPOD®-derived data, MRI identified a 5.8% greater whole-body volume in the PsD group compared to BMI/ethnicity-matched controls. Furthermore, MRI segmentation revealed that the PsD group had a 5.9% greater whole-body VAT volume and a 13.6% greater VAT/SAT ratio compared to BMI/ethnicity-matched controls (*P* < 0.01), illustrated according to PsD disease activity in Fig. 1C and D. Figure 2 depicts overlay of fat segmentation using FatSegNet (PsD vs. control)

### Body composition and associations with PsD disease activity

Based on data derived from ADP, participants with active PsD had an 8.3% lower body fat percentage compared to those with inactive disease; however, this effect was offset by higher whole-body mass, fat mass and fat-free mass.

### Correlations between body composition and metabolic indices

Between the subgroups of PsD (including PsO and PA), both disease activity levels and ADP-derived measures of body composition were equivalent (Table 4). ADP-derived total body mass and total body fat each negatively correlated with serum adiponectin, respectively: unstandardised *ꞵ* − 0.946 (95% CI − 1.665, − 0.226, *P* = 0.001); unstandardised *ꞵ* − 0.473 (95% CI − 0.850, − 0.096, *P* = 0.02). None of the other ADP-derived body composition measures independently associated with PsD disease activity. For the MRI-based data, whole-body VAT and whole-body VAT/SAT showed strong associations with independent variables (age, female sex, PsD group and adiponectin). Older participants had greater whole-body VAT: unstandardised *ꞵ* 66.348 (95% CI 4.112, 128.584, *P* = 0.04) and VAT/SAT: unstandardised *ꞵ* (0.012, 95% CI − 0.000, 0.024, *P* = 0.05). Furthermore, whole-body VAT and VAT/SAT positively associated with PsD disease activity and, as expected, negatively associated with female sex and serum adiponectin.

**Table 4:** Associations between body composition measures and disease variables in patients with PsO and PsA

Variables	Univariable analysis			Multivariable analysis		
	*ꞵ*	95% CI	*P*-value	*ꞵ*	95% CI	*P*-value
Body mass (kg)						
Age (per 5 years)	0.227	− 1.903, 2.357	0.832	0.415	− 1.656, 2.487	0.402
Female sex	− 7.948	− 18.583, 2.687	0.140	− 5.119	− 15.107, 4.869	0.309
Psoriatic group	2.297	− 8.025, 12.618	0.658	1.498	− 8.450, 11.447	0.764
Overall activity	12.669	− 2.180, 2.687	0.91			
HOMA-IR	2.270	0.740, 3.799	*0.004*	1.392	− 0.231, 3.016	0.091
Adiponectin (per 500)	− 1.206	− 1.847, − 0.565	< *0.001*	− 0.946	− 1.665, − 0.226	*0.011*
				*R*^2^ = 0.258		
Body fat (%)						
Age (per 5 years)	0.721	− 0.592, 2.035	0.276	1.088	0.02, 2.174	0.050
Female sex	13.942	8.273, 19.611	< *0.001*	15.817	10.580, 21.054	< *0.001*
Psoriatic group	− 0.100	− 6.539, 6.339	0.975	− 0.294	− 5.510, 4.922	0.910
Overall activity	− 3.240	− 12.215, 5.735	0.466			
HOMA-IR	1.068	0.85, 2.051	*0.034*	0.700	− 0.152, 1.551	0.105
Adiponectin (per 500)	− 0.473	− 0.850, − 0.096	0.101	− 0.473	− 0.850, − 0.096	*0.015*
				*R*^2^ = 0.474		
Fat-free mass (kg)						
Age (per 5 years)	− 0.089	− 1.264, 1.087	0.881	− 0.296	− 1.188, 0.596	0.508
Female sex	− 16.430	− 20.566, − 12.295	< *0.001*	− 15.951	− 20.253, − 11.650	< *0.001*
Psoriatic group	2.850	− 2.805, 8.505	0.317	0.399	− 3.886, 4.683	0.853
Overall activity	6.079	− 2.422, 14.580	0.154			
HOMA-IR	0.337	− 0.564, 1.238	0.457	0.032	− 0.667, 0.731	0.928
Adiponectin (per 500)	− 0.403	− 0.783, − 0.023	*0.038*	− 0.207	− 0.517, 0.103	0.187
				R^2^ = 0.548		
WB vol (cm^3^)						
Age (per 5 years)	329.672	− 221.020, 880.364	0.236	341.554	− 137.047, 820.155	0.158
Female sex	− 636.001	− 3474.652, 2202.649	0.655	493.051	− 1829.215, 2815.318	0.672
Psoriatic group	1684.112	− 1002.446, 4370.690	0.214	339.799	− 1982.724, 2662.322	0.770
Overall activity	1920.658	− 1866.044, 5707.360	0.307			
HOMA-IR	851.247	485.799, 1216.696	< *0.001*	562.705	186.934, 938.476	*0.004*
Adiponectin (per 500)	− 288.537	− 523.101, − 201.594	< *0.001*	− 270.561	− 454.790, − 122.284	*0.001*
				*R*^2^ = 0.420		
WB VAT (cm^3^)						
Age (per 5 years)	97.200	27.481, 166.920	0.007	66.348	4.112, 128.584	*0.037*
Female sex	− 511.662	− 865.631, − 157.693	0.05	− 329.301	− 631.283, − 27.319	*0.033*
Psoriatic group	659.706	341.585, 977.828	< *0.001*	437.101	135.086, 739.117	*0.005*
Overall activity	108.348	− 512.205, 728.900	0.723			
HOMA-IR	74.192	20.413, 127.971	0.008	35.395	− 13.470, 84.259	0.171
Adiponectin (per 500)	− 30.323	− 53.960, − 6.686	0.013	− 21.559	− 43.178, 0.060	*0.050*
				*R*^2^ = 0.449		
WB VAT/SAT						
Age (per 5 years)	0.012	− 0.004, 0.033	0.203	0.012	− 0.000, 0.024	*0.050*
Female sex	− 0.167	− 0.232, − 0.103	< *0.001*	− 0.140	− 0.200, − 0.080	< *0.001*
Psoriatic group	0.127	0.061, 0.194	< *0.001*	− 0.081	− 0.021, − 0.140	*0.009*
Overall activity	0.041	− 0.093, 0.175	< *0.001*			
HOMA-IR	0.006	− 0.005, 0.018	0.280	0.002	− 0.08, 0.012	0.693
Adiponectin (per 500)	− 0.002	− 0.009, 0.001	0.146	− 0.002	− 0.007, 0.002	0.280
				*R*^2^ = 0.491		

*HOMA-IR* Homeostatic Model Assessment for Insulin Resistance, *SAT* subcutaneous adipose tissue, *VAT* visceral adipose tissue, *WB* whole-body

Significant values are in italics

### Associations between body composition and physical activity, diet and quality of life

Correlations between body composition and physical activity, quality of life and metabolic indices are shown in Supplementary Table 2. As shown, body mass, total body fat, whole-body volume and whole-body VAT correlated with higher fasting serum triglyceride levels and cholesterol:HDL ratio. Within the PsD group, those with a higher MRI-derived body volume had a higher HAQ score (Pearson correlation = 0.499) and a lower MDA score (Pearson correlation =  − 0.685).

## Discussion

The premise of this pilot study was to understand in more depth body composition in psoriatic disease, particularly its phenotypic and metabolic associations. Several aspects of body composition, specifically, the amount and distribution of body fat and lean mass, are now understood to be independent health predictors in adults and may form an important part of the ongoing clinical assessment of patients with psoriatic disease.

As expected, patients represented the whole spectrum of psoriatic disease; those with chronic plaque psoriasis and concomitant psoriatic arthritis being by far the largest group. In our cohort, women revealed overall lower body mass and volumes yet higher body fat when compared to men, whereas men revealed comparatively higher visceral fat; such characteristic sex differences in body composition have been well established [29, 30]. This female pattern of fat distribution is known to be associated with a more favourable cardiovascular risk at a similar BMI; however, ectopic fat deposition within the abdomen, pericardium and neck is more strongly implicated in women’s adverse cardiovascular risk than that of men. Sex dimorphism in the heritability suggests that female fat distribution may be more genetically affected than males, and biological pathways are differentially involved in the determination of body fat distribution [31]. The molecular mechanism for this sex dimorphism may also be beyond the modulation of sex hormones [32].

Regarding the psoriatic group, they demonstrated adverse body composition profiles across the board, including higher body mass, whole-body volume, subcutaneous and visceral fat. This relationship could not be explained by lifestyle factors such as physical activity levels, diet or smoking; ironically, patients with PsD were seen to be maintaining as much vigorous exercise as their healthy counterparts. More patients in the PsA subgroup had active disease, as measured by MDA, and demonstrated higher visceral fat, although this effect was not revealed in the purely cutaneous PsO patients, exemplifying the fact that there are often more diverse contributory factors and nuances to disease activity in a PsA population. If we consider the observed dysmetabolism is a consequence of inflammation, it is however, not clear if the underlying psoriasis itself or the visceral adipose tissue is the key player. Moreover, the association between psoriatic disease activity and MRI-derived visceral fat distribution was noted to be starker in men than women. This finding could have important consequences when assessing individuals’ composite metabolic risk and its potential impact on efficacy of systemic therapies.

The data indicated correlations between patients’ unfavourable body composition profiles, disease activity and cardiometabolic measures of the archetypal metabolic syndrome, specifically, cholesterol:HDL cholesterol ratio and triglycerides. Epidemiological studies have tended to focus on weight or BMI to define obesity rather than altered body composition. Interestingly, there is conflicting data on the association between psoriasis severity, such as PASI, and body composition parameters, indicating that a causal link is by no means definitive. Previous studies have alluded to a dose–response relationship between psoriasis severity and metabolic syndrome [33], supported by translational studies showing T-helper cell cytokine upregulation in the blood and skin of psoriasis patients, leading to effects on lipid metabolism and insulin resistance [34].

Quantification and accurate localisation of various adipose tissue depots is of high research interest in chronic disease particularly those of an inflammatory nature. The last decade has seen an impetus in the development and validation of new modalities for the assessment of body composition. The ratio between abdominal VAT/SAT has been identified as an independent predictor of death and coronary events, irrespective of cardiovascular risk factors and the presence of coronary artery disease [35]. Similarly, quantification of VAT volume and VAT/SAT volume ratio by MRI has been found to be a reproducible biomarker associated with cardiometabolic risk factors in subjects with impaired glucose metabolism [36]. Whole-body fat quantities derived from a continuously moving table Dixon sequence MRI have shown high reproducibility of results ratifying its potential for future research studies [37]. Moreover, the accuracy of this method and the high reproducibility of results indicate its potential for clinical applications.

FatSegNet is a novel, fully automated deep learning pipeline that utilises a competitive dense fully convolutional network (CDFNet) architecture to localise VAT and SAT on abdominal Dixon MR images. It can accurately segment visceral and subcutaneous adipose tissue inside a consistent anatomically defined abdominal region and has been shown to outperform manual rating of VAT (0.850 vs. 0.788) and SAT (0.975 vs. 0.982). In accordance with previous studies on small datasets [38, 39], our data showed a sex- and age-specific difference of VAT accumulation, wherein men and older patients were more likely to have higher VAT compared to women and younger patients. This method of fat segmentation is efficient, well-tolerated and reliable [26]. Furthermore, FatSegNet has been shown to go one better than other architectures employed in body composition mapping and, in our case, proved to be far more informative than the technique of air displacement plethysmography for demonstrating important phenotypic and metabolic differences between psoriatic patients and controls.

Some studies have employed manual techniques for the assessment of visceral fat in chronic disease, such as the visceral adiposity index (VAI), a gender-specific empirical mathematical model based on simple anthropometric (BMI and waist circumference) and functional parameters (TG and HDL) and indicative of fat distribution and function [40]. There is, however, a distinct lack of prospective evidence showing VAI to have a prognostic role in CV risk, especially in the context of inflammatory disease, and given the relative simplicity of MRI-based assessment, we suggest that the VAT and VAT/SAT could become an easy tool for the evaluation of adipose tissue dysfunction and its associated cardiometabolic risk in various patient populations, for example, those at risk for a metabolic syndrome.

Studies of spondyloarthritis, RA and psoriasis have reported a reduced efficacy, drug survival and adherence to tumour necrosis factor inhibitors (TNFis) in obese patients [41–46]. There are also data linking the human TNF receptor fusion protein, Etanercept, with weight gain [47]. In PsD, the impact of obesity on TNFis remains unclear since available studies are small, present diverging results and lack long-term follow-up data. Treatment with anti-IL-12/23 inhibitors has been associated with more favourable body composition profiles than TNFis, findings which parallel previous observations of increases in BMI seen with this class of drug [48, 49]. IL-17, one of the key proinflammatory cytokines in psoriasis, mechanistically links inflammation with insulin resistance and adipocyte dysfunction [50]. IL-17A-producing cells are thought to be pathogenic in driving inflammation in obesity and progression of obesity-related inflammatory diseases, suggesting that causality between psoriasis and adipogenesis is likely to be bidirectional [51]. From this perspective, there are likely to be therapeutic implications of targeting proinflammatory factors such as IL-17 or IL-12/23 in metabolic dysfunction associated with psoriatic disease. A recent prospective, open-label study (Immune Metabolic Associations in Psoriatic Arthritis) evaluated the effect of the phosphodiesterase-4 (PDE4) inhibitor apremilast on body weight and composition and observed weight loss, principally abdominal subcutaneous fat, and improvement in psoriatic disease activity independent of weight change [52]. These findings were paralleled by the results of the VIP-A trial, a single-arm, open-label, interventional, non-randomised clinical trial, in which CT imaging and laboratory outcomes were measured in patients taking apremilast. Patients showed reductions in visceral and subcutaneous fat as well as beneficial effects on cardiometabolic biomarkers [53]. Considering this, we postulate that further individualised treatment strategies based on multimodal insight into adverse metabolic profiles and biomarkers, such as high visceral fat, may improve outcomes and overall care of psoriatic patients. An automated model of fat segmentation—being less expensive and time-consuming than manual segmentation—could facilitate future research of similar patients using large population-based cohorts.

### Strengths

To our knowledge, this is the first time that a deep learning application for MRI-derived body composition, especially that of VAT and its metabolic significance, has been studied in psoriatic disease and compared to matched controls. We have reported on a novel, automated method for image acquisition and validated its functionality in a clinical cohort with chronic inflammatory disease.

### Limitations

We are aware that this is a pilot study and as such will likely need to be repeated on a wider scale. The cross-sectional nature of the study confers challenges with determining causal relationships. The relatively small sample size of patients and controls and diverse age range will also have hampered the data analysis and ability to draw certain conclusions. We believe that further research in this field will enhance the validity of our results whilst keeping a realistic view of the expected numbers of patients that can be recruited to a similar study.

## Conclusions

This study shows that visceral adipose [a more metabolically active depot] can provide additional value to current measures of obesity such as BMI and waist-to-hip ratio and contribute to the unfavourable metabolic dysfunction seen in psoriatic disease. Our data support the concept that defined body composition changes are independent of the customary metabolic syndrome and that disease activity, not just occurrence of psoriasis, is unequivocally more than skin-deep and seems to correlate with underlying visceral fat. Assessment of abdominal adiposity through MRI-based segmentation, specifically, that of VAT and VAT/SAT volume ratio, may provide a more accurate evaluation of adipose dysfunction and complement information obtained through conventional measurements. These indices may serve as useful biomarkers of an adverse inflammatory state seen in psoriatic disease.

A deep learning algorithm employing automated MRI-determined fat segmentation shows good association with disease activity and metabolic dysfunction, findings that cannot be simulated by anthropometric assessment or air displacement plethysmography. MR imaging and automated fat analysis could serve as a prototype for the valuable assessment of the metabolic and body composition effects of targeted therapies in complex inflammatory disease. It is possible that such novel systems will eventually supplement less sophisticated bedside measurements and influence key aspects of risk assessment, prognostication and management in patients with psoriatic disease. Further prospective studies are needed to confirm these preliminary results.

## Supplementary Information

**Additional file 1:  Supplementary Table 1.** Variables measured.

**Additional file 2: Supplementary Table 2.** Correlation between body composition and physical activity, quality of life and metabolic indices.

## Data Availability

The data underlying this article will be shared on reasonable request to the corresponding author.

## Abbreviations

AAT: Abdominal adipose tissue
ADP: Air displacement plethysmography
BIA: Bioimpedance analysis
BMI: Body mass index
BMR: Basal metabolic rate
BSA: Body surface area
CASPAR: Classification Criteria for Psoriatic Arthritis
CDFNet: Competitive dense fully convolutional network
CRP: C-reactive protein
CT: Computed tomography
CV: Cardiovascular
DAPSA: Disease Activity in Psoriatic Arthritis
DLQI: Dermatology Life Quality Index
DXA: Dual energy X-ray absorptiometry
ESR: Erythrocyte sedimentation rate
HAQ: Health Assessment Questionnaire
HbA1c: Haemoglobin A1c
HDL: High-density lipoprotein
HMRU: Human Metabolism Research Unit
HOMA-IR: Homeostasis Model Assessment for Insulin Resistance
IL: Interleukin
IQR: Interquartile range
IPAQ: International Physical Activity Questionnaire
LEI: Leeds Enthesitis Index
LDL: Low-density lipoprotein
MDA: Minimal disease activity
MET: Metabolic equivalent of task
MetS: Metabolic syndrome
MRI: Magnetic resonance imaging
PASI: Psoriasis Area Severity Index
PsA: Psoriatic arthritis
PsD: Psoriatic disease
PsO: Psoriasis
SAT: Subcutaneous adipose tissue
SD: Standard deviation
SPARCC: Spondyloarthritis Research Consortium of Canada
T3: Triiodothyronine
T4: Thyroxine
TSH: Thyroid-stimulating hormone
TG: Triglycerides
TNF-α: Tumour necrosis factor alpha
TNFi: Tumour necrosis factor inhibitor
UHCW: University Hospitals Coventry and Warwickshire
VAI: Visceral adiposity index
VAT: Visceral adipose tissue
VLDA: Very low disease activity
WB: Whole-body
WC: Waist circumference
