# Correction: More than skin‑deep: visceral fat is strongly associated with disease activity, function and metabolic indices in psoriatic disease

**DOI:** 10.1186/s13075-023-03102-x

**Published:** 2023-07-04

**Authors:** Tim Blake, Nicola J. Gullick, Charles E. Hutchinson, Abhir Bhalerao, Sarah Wayte, Andrew Weedall, Thomas M. Barber

**Affiliations:** 1 Department of Rheumatology, University Hospitals Coventry and Warwickshire, Clifford Bridge Road, Coventry, CV2 2DX UK; 2 Warwick Medical School, University of Warwick, Coventry, CV4 7HL UK; 3 Division of Biomedical Sciences, Warwick Medical School, Clinical Sciences Research Laboratories, University Hospitals Coventry and Warwickshire, Coventry, CV2 2DX UK; 4 Department of Computer Science, University of Warwick, Coventry, CV4 7EZ UK; 5 Department of Radiology, University Hospitals Coventry and Warwickshire, Coventry, CV2 2DX UK; 6 Radiology Physics, Department of Clinical Physics and Bioengineering, University Hospitals Coventry and Warwickshire, Coventry, CV2 2DX UK; 7 Warwickshire Institute for the Study of Diabetes, Endocrinology and Metabolism, University Hospitals Coventry and Warwickshire, Coventry, CV2 2DX UK

**Correction: Arthritis Res Ther 25, 108 (2023)**

**https://doi.org/10.1186/s13075-023-03085-9**

Following publication of the original article [1], the authors reported an error in the author group. The given names are currently presented in abbreviated form and it was requested that the given names be expanded to full.

The author group has been updated above and the original article [1] has been corrected.
