# Supplementary material for: More than skin-deep: visceral fat is strongly associated with disease activity, function and metabolic indices in psoriatic disease
Source: Arthritis Res Ther. 2023 Jun 23;25:108. doi: 10.1186/s13075-023-03085-9 (PMC10288730; doi:10.1186/s13075-023-03085-9)
Supplement: Supplementary file 1 — Additional file 1: Supplementary Table 1. Variables measured. [file 13075_2023_3085_MOESM1_ESM.docx]

**Additional file 1: Supplementary Table 1. Variables measured**

| **Variables** | **Total Psoriasis**  (n = 30) | **With PsA**  (n = 22) | **Without PsA**  (n = 8) | **Controls**  (n = 30) | ***P*-value** |
| --- | --- | --- | --- | --- | --- |
| MET (mins/week)  Mean (SD)  Median (IQR) | 4480.1 (4327.55)  3637 (3888) | 4390.68 (4535.1)  3485 (3888) | 4726 (3973.58)  3898 (5212.5) | 4025.84 (2488.51)  2878.5 (3085.5) | 0.620* |
| Vigorous activity (mins/week)  Mean (SD)  Median (IQR) | 95.6 (183.95)  0 (60) | 102.41 (205.3)  0 (60) | 76.88 (114.92)  22.5 (135) | 167.33 (229.72)  127.5 (180) | 0.069** |
| Sitting time (mins/week)  Mean (SD)  Median (IQR) | 2686 (1505.96)  2310 (2100) | 2863.64 (1393.8)  2520 (2100) | 2197.5 (1787.65)  1680 (3120) | 2926 (871.86)  3150 (840) | 0.738* |
| BMR (calories/day)  Mean (SD)  Median (IQR) | 1627.08 (348.43)  1570.92 (586.56) | 1691.32 (373.52)  1639.56 (599.04) | 1450.41 (189.10)  1424.28 (313.56) | 1538.16 (334.13)  1486.68 (393.12) | 0.317* |
| CRP (mg/L)  Mean (SD)  Median (IQR) | 4.83 (8.83)  1.5 (6) | 5.86 (10.04)  3.5 (9) | 2.0 (2.78)  0.0 (5) | 2.17 (3.88)  0 (3) | 0.143** |
| ESR (mm/hr)  Mean (SD)  Median (IQR) | 11.00 (10.52)  7.5 (15) | 11.64 (10.89)  8 (16) | 9.25 (9.91)  6.5 (8.5) | 11.87 (10.36)  8.5 (13) | 0.748* |
| TSH (mU/L)  Mean (SD)  Median (IQR) | 2.08 (0.89)  1.96 (0.8) | 2.22 (0.72)  2.14 (0.72) | 1.73 (1.24)  1.37 (0.96) | 1.87 (0.85)  1.78 (1.02) | 0.313** |
| T3 (pmol/L)  Mean (SD)  Median (IQR) | 4.85 (0.63)  4.9 (1) | 4.98 (0.64)  5.15 (0.9) | 4.5 (0.49)  4.3 (0.7) | 4.64 (0.50)  4.6 (0.9) | 0.158* |
| T4 (pmol/L)  Mean (SD)  Median (IQR) | 15.10 (2.13)  15.1 (2.3) | 15.23 (2.35)  15.2 (2.8) | 14.65 (1.38)  14.35 (2.4) | 15.61 (2.39)  15.35 (2.8) | 0.387* |
| Total cholesterol (mmol/L)  Mean (SD)  Median (IQR) | 5.45 (1.00)  5.4 (1.3) | 5.35 (0.89)  5.45 (1.5) | 5.71 (1.28)  5.25 (1.45) | 5.16 (1.09)  5.35 (1.5) | 0.271* |
| Triglycerides (mmol/L)  Mean (SD)  Median (IQR) | 1.37 (0.60)  1.2 (0.9) | 1.33 (0.51)  1.2 (0.7) | 1.49 (0.83)  1.4 (1.2) | 1.26 (0.81)  1.0 (0.9) | 0.701* |
| HDL-C (mmol/L)  Mean (SD)  Median (IQR) | 1.42 (0.37)  1.4 (0.3) | 1.40 (0.42)  1.35 (0.4) | 1.48 (0.17)  1.45 (0.2) | 1.43 (0.37)  1.4 (0.6) | 0.917* |
| LDL-C (mmol/L)  Mean (SD)  Median (IQR) | 3.41 (0.90)  3.45 (0.9) | 3.35 (0.91)  3.5 (1.1) | 3.56 (0.89)  3.1 (1.3) | 3.16 (0.91)  3.25 (1.5) | 0.289* |
| HbA1c (mmol/mol)  Mean (SD)  Median (IQR) | 36.97 (7.76)  36.00 (5) | 35.55 (7.77)  35 (5) | 40.88 (6.64)  39 (7) | 34.67 (2.92)  35.00 (5) | 0.134* |
| Fasting glucose (mmol/L)  Mean (SD)  Median (IQR) | 5.83 (1.22)  5.5 (1.1) | 5.77 (1.25)  5.5 (0.7) | 5.99 (1.23)  5.95 (1.3) | 5.17 (0.41)  5.15 (0.6) | 0.007** |
| Fasting insulin (pmol/L)  Mean (SD)  Median (IQR) | 84.37 (66.27)  66.00 (74.5) | 88.91 (74.14)  67 (85) | 71.88 (38.23)  59 (44.5) | 76.37 (82.15)  42.5 (48) | 0.339* |
| HOMA-IR  Mean (SD)  Median (IQR) | 3.56 (3.32)  2.5 (2.7) | 3.77 (3.66)  2.55 (2.9) | 2.96 (2.23)  2.35 (2) | 2.63 (3.00)  1.4 (1.6) | 0.111* |
| Adiponectin (µg/ml)  Mean (SD)  Median (IQR) | 7100.08 (3233.97)  6837.6 (4662.3) | 7242.41 (3520.87)  7110.6 (3954.6) | 6708.7 (2428.99)  6498.15 (4295.6) | 7630.32 (4050.04)  6655.5 (5407.7) | 0.289* |
| Body Mass (kg)  Mean (SD)  Median (IQR) | 82.99 (19.80)  83.35 (28.6) | 85.4 (18.5)  84.9 (23.6) | 76.34 (23.09)  79.6 (40.05) | 73.9 (20.14)  73.9 (29.5) | 0.328* |
| Body Fat (%)  Mean (SD)  Median (IQR) | 36.85 (11.47)  37.85 (13.6) | 35.00 (10.57)  36 (14.6) | 42.09 (12.96)  42.6 (8.2) | 36.99 (13.37)  40.85 (21.9) | 0.965* |
| Fat Mass (kg)  Mean (SD)  Median (IQR) | 32.95 (14.85)  31.4 (15.1) | 31.17 (13.66)  29.5 (14.6) | 37.84 (17.80)  37.3 (20) | 31.14 (16.52)  28.2 (25.5) | 0.329* |
| Fat Free Mass (kg)  Mean (SD)  Median (IQR) | 52.15 (11.17)  50.2 (18.8) | 54.21 (11.97)  52.55 (19.2) | 46.49 (6.06)  45.65 (10.05) | 49.3 (10.71)  47.65 (12.6) | 0.822* |
| WB volume (cm^3^)  Mean (SD)  Median (IQR) | 21371.85 (4730.86)  22259.19 (6308.91) | 21540.67 (4704.27)  21695.36 (6521.87) | 20841.27 (153.33)  23166.51 (6410.75) | 19687.73 (5528.04)  19495.20 (8440.43) | 0.107* |
| WB SAT (cm^3^)  Mean (SD)  Median (IQR) | 6133.47 (2502.11)  6533.14 (3175.75) | 6031.53 (2521.69)  5721.78 (2596.87) | 6453.88 (2608.01)  7134.48 (4356.81) | 6557.67 (3105.32)  6254.23 (5237.26) | 0.283* |
| WB VAT (cm^3^)  Mean (SD)  Median (IQR) | 1202.45 (761.99)  1202.82 (1142.86) | 1298.09 (820.88)  1346.90 (1559.67) | 901.86 (460.64)  997.40 (502.44) | 542.74 (413.37)  390.90 (564.47) | **<0.001*** |
| WB AAT (cm^3^)  Mean (SD)  Median (IQR) | 7335.92 (2594.27)  8027.61 (3440.96) | 1346.90 (820.88)  1346.90 (1559.67) | 7355.74 (2934.18)  8337.30 (4734.60) | 7100.41 (3280.31)  6760.10 (4634.84) | 0.380* |
| WB VAT/AAT  Mean (SD)  Median (IQR) | 0.17 (0.11)  0.15 (0.17) | 0.18 (0.11)  0.19 (0.19) | 0.11 (0.05)  0.11 (0.07) | 0.08 (0.06)  0.06 (0.08) | **<0.001*** |
| WB VAT/SAT  Mean (SD)  Median (IQR) | 0.22 (0.17)  0.18 (0.24) | 0.25 (0.18)  0.24 (0.27) | 0.13 (0.07)  0.13 (0.09) | 0.09 (0.08)  0.07 (0.09) | **<0.001*** |

*Independent T-test: psoriatic group vs controls; Significant values are in bold.

AAT: abdominal adipose tissue; BMR: basal metabolic rate; CRP: C-reactive protein; ESR: erythrocyte sedimentation rate; HbA1c: haemoglobin A1c; HDL: high-density lipoprotein; HOMA-IR: Homeostatic Model Assessment for Insulin Resistance; IQR: interquartile range; LDL: low-density lipoprotein; MET: metabolic equivalent of task; SAT: subcutaneous adipose tissue; SD: standard deviation; T3: triiodothyronine; T4: thyroxine; TSH: thyroid stimulating hormone; VAT: visceral adipose tissue; WB: whole-body.
