# Supplementary material for: More than skin-deep: visceral fat is strongly associated with disease activity, function and metabolic indices in psoriatic disease
Source: Arthritis Res Ther. 2023 Jun 23;25:108. doi: 10.1186/s13075-023-03085-9 (PMC10288730; doi:10.1186/s13075-023-03085-9)
Supplement: Supplementary file 2 — Additional file 2: Supplementary Table 2. Correlation between body composition and physical activity, quality of life and metabolic indices. [file 13075_2023_3085_MOESM2_ESM.docx]

**Additional file 2: Supplementary Table 2. Correlation between body composition and physical activity, quality of life and metabolic indices**

| **Characteristics** | **Vig Ac** | **MET (mins/week)** | **BMR** | **VAS** | **HAQ** | **PASI** | **MDA** | **Trigs** | **TC:HDL** |
| --- | --- | --- | --- | --- | --- | --- | --- | --- | --- |
| **Body mass** | 0.108 | -0.097 | -0.001 | 0.421* | 0.470** | 0.157 | -0.690 | 0.387** | 0.399** |
| **Body fat** | -0.072 | -0.062 | 0.002 | 0.157 | 0.170 | -0.28 | -0.300 | 0.338** | 0.111 |
| **Fat free mass** | 0.131 | -0.112 | 0.010 | 0.234* | 0.281 | 0.059 | -0.359 | 0.092 | 0.294* |
| **WB volume** | 0.072 | -0.117 | -0.999 | 0.024* | 0.499** | 0.057 | -0.685** | 0.486** | 0.450** |
| **WB VAT** | -0.190 | -0.269* | -0.494** | -0.053 | 0.310 | -0.236 | 0.066 | 0.287* | 0.276* |
| **WB VAT/SAT** | -0.094 | -0.245 | -0.462** | -0.049 | 0.176 | -0.273 | 0.131 | 0.147 | 0.201 |

*Correlation is significant at the 0.05 level, **correlation is significant at the 0.01 level.

BMR: basal metabolic rate; HAQ: Health Assessment Questionnaire; MDA: minimal disease activity; MET: metabolic equivalent of task; PASI: Psoriasis Area and Severity Index; TC:HDL: total cholesterol/high-density lipoprotein cholesterol; SAT: subcutaneous adipose tissue; VAS: Visual Analogue Scale, VAT: visceral adipose tissue; WB: whole-body.
